# Central Glossary

**Published:** 2010

Adaptive immune response: Second line of the immune response that is specific to a given foreign molecule or *pathogen* and leads to an “immunological memory” after the first response to the molecule or *pathogen*.
Additive: The effect when two or more agents act together to achieve a result that is the sum of the effects achieved by the individual agents.
AIDS-defining illness or event: Any one of a group of illnesses presence of which indicates that a patient’s HIV infection has progressed to the acquired immune deficiency syndrome (AIDS); these conditions include, among others, yeast infections of the airways (i.e., candidiasis), Kaposi’s sarcoma, certain viral infections, tuberculosis, and certain cancers.
Airway obstruction: Respiratory problem in which the small airways (bronchioles) show increased resistance, thereby reducing the amount of air inhaled in each breath and, consequently, the amount of oxygen that can be taken up into the body.
Alveolus (pl. alveoli): Tiny airsac found in the mammalian lungs at the end of the air passages (bronchioles) that make up most of the lung tissue and which are the site of the oxygen and carbon dioxide exchange in the lungs; a human lung contains about 150 million alveoli.
Anterior: Towards the front (e.g., the anterior region of the brain is located toward the front of the head).
Antibody: A protein produced by the body’s immune system to recognize and fight infectious organisms and other foreign substances that enter the body. Each antibody is specific to a particular piece of an infectious organism or other foreign substance. Antibodies develop after the first exposure to a substance.
Antigen: Any substance considered foreign to the body that can stimulate the body to produce *antibodies* against it.
Antiretroviral: A drug that interferes with the ability of a retrovirus, such as HIV, to make more copies of itself.
Antiretroviral therapy (ART): Medications for the treatment of infection by retroviruses and primarily is used to treat HIV.
Apoptosis: Cell death in which the affected cell participates by activating a cascade of biochemical reactions that lead to death; also known as programmed cell death or cell suicide.
Asthma: Chronic inflammation of the lungs and airways (bronchi and bronchioles), leading to a reversible narrowing of the airways and breathing difficulties.
Atrial fibrillation: A type of abnormal heart rhythm (i.e., arrhythmia) that involves the upper chambers (i.e., atria) of the heart; characterized by quivering rather than coordinated contraction of the muscles of the atria.
Axon: The long, thin fiber protruding from a nerve cell that carries integrated nerve signals in the form of electrical signals to other nerve cells.
B-lymphocyte: Also known as a B-cell. Infection-fighting white blood cell that develops in the bone marrow and spleen. B-lymphocytes produce antibodies. In people with HIV, the ability of B-lymphocytes to do their job may be damaged.
Bacteremia: The presence of viable bacteria in the blood.
Bronchiectasis: Disease of the airways characterized by a localized, irreversible widening (dilation) of a part of the airways; the affected bronchi and bronchioles are dilated, inflamed and collapse easily, resulting in reduced airflow; usually caused by bacterial infections.
Basal ganglia: A group of *nuclei* in the brain interconnected with the cerebral *cortex*, thalamus and brainstem. Mammalian basal ganglia are associated with a variety of functions: motor control, cognition, emotions, and learning.
Cachexia: A syndrome characterized by weight loss, muscle *atrophy*, fatigue, weakness, and loss of appetite that cannot be reversed by increased nutrition.
Cardiovascular disease: Generic term for diseases that involve the heart or blood vessels.
Caudate nucleus: A nucleus located within the *basal ganglia* of the brains of many animal species. The caudate, originally thought to primarily be involved with control of voluntary movement, is now known to be an important part of the brain’s learning and memory system.
CD4+ T-cell: A type of *lymphocyte*; the main target in the body for infection by HIV.
CD8 cell: Also called a cytotoxic T-lymphocyte (CTL), killer T-cell, or suppressor T-cell. A type of white blood cell that is able to identify and kill cells infected with bacteria, viruses, or other foreign invaders.
Cell-mediated immunity: Immune protection provided by the direct action of immune cells. With this type of immune protection, the response to infectious micro-organisms is performed by specific cells—such as CD8 cells, macrophages, and other white blood cells—rather than by antibodies. The main role of cell-mediated immunity is to fight viral infections.
Centrum semiovale: The white matter found beneath the cerebral *cortex*.
Cerebellar: Pertaining to the cerebellum, a region of the brain that controls sensory perception and motor functions.
Cholesterol: A waxy steroid *metabolite* found in cell membranes and transported in the blood of all animals; high levels of cholesterol in the blood may contribute to heart disease.
Chronic obstructive pulmonary disease (COPD): Term referring to chronic bronchitis and *emphysema*, two conditions in which the airways become narrowed, resulting in chronic breathing difficulties; in contrast to *asthma*, the narrowing of the airways is only poorly reversible and typically becomes progressively worse.
Confidence interval (CI): Statistical measure of the reliability of an estimate; the range of values that is likely to include the actual value of the variable being estimated.
Congestive heart failure: Inability of the heart to supply sufficient blood flow to meet the body’s need, resulting in shortness of breath, fluid accumulation in the blood vessels and tissues, or coughing; can be caused by *myocardial infarction*.
Coronary heart disease: Failure of the blood vessels of the heart muscle to supply adequate blood and nutrients to the heart muscle and surrounding tissues.
Corpus callosum: The largest connective pathway in the human brain. It is made of nerve fibers that connect the left and right sides (hemispheres) of the brain.
Cortex: The outer layer of the largest part of the brain (i.e., cerebrum) that plays a key role in memory, attention, perceptual awareness, thought, language, and consciousness.
Cumulative: The combined effect when two or more agents act together, can be *additive* or *synergistic*.
Cytokines: A family of molecules, produced primarily by cells of the immune system, that regulate cellular interactions and other functions. Many cytokines play important roles in initiating and regulating inflammation.
Cytotoxic T-lymphocyte: Also known as killer T-cell, or suppressor T-cell. A type of white blood cell that is able to identify and kill cells infected with bacteria, viruses, or other foreign invaders.
Diffusion tensor imaging (DTI): A type of *magnetic resonance imaging (MRI)* that allows measurement of the restricted movement of water molecules in tissues, enabling researchers to analyze the structural integrity particularly of *white matter* tracts.
Dyslipidemia: Any abnormality in the amount of lipids (e.g., *cholesterol* or fat) in the blood.
Edema: Abnormal accumulation of fluid in a tissue or in one or more body cavities.
Electron transport chain: The electron transport system (also known as respiratory chain) located in the *mitochondria*, in which electrons released by NADH are passed on to a series of other molecules in successive reactions that provide enough energy to drive the synthesis of ATP molecules.
Emphysema: Chronic lung disorder characterized by loss of elasticity of the lung tissue. In these patients, the small airways to collapse during forced exhalation. As a result, airflow out of the lungs is impeded and air becomes trapped in the lungs. Symptoms include shortness of breath on exertion, and an expanded chest.
Fibrosis: The formation of scar tissue.
Forced expiratory volume in 1 second (FEV_1_): A measure of lung function; the volume of air that can be exhaled in 1 second; to determine the FEV_1_, the patient is asked to inhale deeply and then to exhale as hard as he or she can, and the volume that is exhaled in the first second is measured.
Fulminant hepatic failure: A potentially reversible type of acute liver failure, characterized by rapid progression from early symptoms (e.g., jaundice) to advanced stages characterized by confusion, altered level of consciousness and coma (i.e., encephalopathy).
Genu: The *anterior* end of the *corpus callosum.*
Gray matter: A generic term for a collection of neuronal cell bodies in the central nervous system.
Hazard ratio: The effect of an explanatory variable on the hazard or risk of an event occurring; it can be considered an estimate of the *relative risk*.
Highly active anti-retroviral therapy (HAART): Treatment approach for HIV-1 infection that involves the combination of several active drugs (antiretroviral drugs); involves a relatively complex and strict treatment regimen and potentially severe side effects, which may limit patient compliance with the treatment.
Humoral immunity: Term used to describe the body’s antibody-based immune response, as opposed to its cell-based immune response (cell-mediated immunity). Immune cells called B-cells produce antibodies against foreign invaders.
Hypertension: Elevated blood pressure.
Inferior: Located below (i.e., toward the feet of) another structure.
Innate immune response: Initial immediate immune response that is not specific to a certain foreign molecule or *pathogen*.
Insulin resistance: Condition in which normal amounts of insulin are inadequate to produce a normal insulin response from fat, muscle, and liver cells.
Kaposi’s sarcoma: A tumor caused by a human herpesvirus that manifests as dark blotches or lesions on the skin as well as in the mouth, gastrointestinal tract, and respiratory tract; considered a defining illness in AIDS patients.
Lactic acidosis: A condition characterized by the accumulation of lactic acid in bodily tissues.
Limbic system: A group of brain structures that together control such functions as emotion, behavior, and long-term memory.
Lipoatrophy: The localized loss of fat tissue; typically occurs as the result of certain injections or as an adverse reaction to certain antiretroviral drugs.
Lipodystrophy: A condition characterized by abnormal or degenerative conditions of the body’s fat tissues.
Lipoprotein: A compound molecule that contains both a protein and a lipid component.
Lymphocyte: A white of type blood cell necessary for the normal immune response; types of lymphocytes include T cells, B cells, and natural killer cells.
Macrophage: A type of immune cell that ingests foreign particles and microorganisms and synthesizes proteins and other substances important in inflammatory responses, including *cytokines*.
Magnetic resonance imaging (MRI): A noninvasive medical imaging technique used to visualize detailed internal structures; allows for differentiation of different soft tissues of the body. MRI uses no radiation but powerful magnetic fields and radio frequency fields that act on the nuclei of hydrogen atoms in the water molecules found in the body.
Metabolite: Intermediary product generated during the metabolism of a particular molecule.
Magnetic resonance spectroscopy (MRS): A noninvasive imaging technique related to *magnetic resonance imaging* (*MRI*) that acts by detecting different chemical nuclei (e.g., phosphorus, carbon, or sodium) within the body in addition to the nuclei of hydrogen atoms.
Mallory body: An inclusion (or body) typically found in the cytoplasm of liver cells of people suffering from alcoholic liver disease.
Microsomal ethanol–oxidizing system (MEOS): An enzyme system involving cytochrome P450 that breaks down alcohol and generates toxic products, such as acetaldehyde and *reactive oxygen species* (*ROS*).
Mitochondria: Structures within cells that generate most of the cells’ energy through the production of adenosine triphosphate, a molecule that provides the energy needed for many key metabolic reactions.
Mitochondrial DNA: DNA molecules found in the *mitochondria* (rather than in the cell nucleus) and encoding more than 30 genes, some of which encode proteins involved in the *electron transport chain.*
Monocyte: Type of *mononuclear* white blood cell; part of the body’s immune system with several roles in the body’s immune response.
Mononuclear cell: White blood cells with a one-lobed nucleus; include *monocytes* and *lymphocytes*.
Myelin sheath: A white fatty material composed chiefly of alternating layers of lipids and *lipoproteins* that encloses the *axons* of most nerve fibers.
Myocardial infarction: Heart attack; disruption of the blood supply to a part of the heart, causing death of heart cells; is a complication of *coronary heart disease.*
*N*-acetyl aspartate (NAA): The second most common compound found in the brain that has several important functions in maintaining normal brain functioning; produces the strongest signal in *magnetic resonance spectroscopy* (*MRS*) analyses of the human brain.
Nucleoside reverse transcriptase inhibitor (NRTI): A class of medications used to treat HIV infection; they act by inhibiting the enzyme reverse transcriptase, which is produced by HIV and related viruses and is needed by viruses to reproduce.
Odds ratio: The ratio of the odds of an event occurring in one group of people to the odds of it occurring in another group.
Opportunistic infection: An infection caused by a *pathogen* that normally does not cause disease in people with a healthy immune system but can cause infection in people with a compromised immune system (e.g., after HIV-1 infection or organ transplant)
Organelle: A specialized subunit in the cell with a specific function (e.g., *mitochondria* are organelles).
Oxidative stress: An imbalance between oxidants (e.g., *reactive oxygen species* [*ROS*]) and antioxidants that can lead to excessive oxidation and cell damage.
Pathogen: Any microorganism (e.g., virus, bacterium, or fungus) that can infect an organism and cause disease in the host.
Periventricular: Referring to a region near the fluid-filled cavities (i.e., ventricles) in the brain.
Phagocytosis: Process by which certain cells (e.g., *macrophages*) engulf a solid particle (e.g., a molecule, bacterium, or virus) and destroy it within the cell.
Pontocerebellar: Referring to a brain system comprising a structure in the brain stem (i.e., the pons) and the cerebellum. The pons relays sensory information between the *cerebellum* and the largest portion of the brain (i.e., cerebrum)
Postural instability: Inability to keep the body in a stable or balanced position.
Posterior: Toward the back (e.g., the posterior region of the brain is located toward the back of the head).
Proteasome: Large protein complexes in the cell that serve to break down and eliminate unneeded or damaged proteins.
Proteasome inhibitor: Agent that inhibits the normal functioning of the *proteasomes*, thereby inducing *apoptosis*; protea-some inhibitors are used in the treatment of certain cancers.
Proton spectrum: The different signals emitted by hydrogen nuclei (i.e., protons) present in different molecules measured during *magnetic resonance spectroscopy* (*MRS*).
Pulmonary hypertension: Elevated blood pressure in the blood vessels connected to or within the lungs, leading to a tightening (vasoconstriction) of those blood vessels and resulting in such symptoms as shortness of breath, dizziness, and fainting.
Quantitative fiber tracking: Computational technique that indirectly analyzes the anatomy of *white matter* fiber systems. It does not actually depict anatomic structures but involves a statistical analysis of the *white matter* integrity as determined by *diffusion tensor imaging* (*DTI*).
Reactive oxygen species (ROS): Highly reactive oxygen– containing free radicals that are generated during oxidative metabolism. ROS can react with and damage lipids, proteins*,* and DNA in cells, causing *oxidative stress*.
Reduced glutathione: An antioxidant that can neutralize *reactive oxygen species* (*ROS*) and other free radicals.
Relative risk: The ratio of the risk that an event (e.g., development of a disease) occurs in people exposed to a given factor (e.g., alcohol consumption) and the risk that the event occurs in people not exposed to the factor.
Resonance: When certain nuclei (e.g., hydrogen, phosphorus, or carbon) are placed in a magnetic field and exposed to radio-frequency waves during *magnetic resonance imaging* (*MRI*) or *magnetic resonance spectroscopy* (*MRS*), they absorb energy from waves with a certain frequency and radiate back this energy with a different frequency; this is called the resonance frequency.
Seroconversion: The process by which a newly infected person develops *antibodies* to HIV. These *antibodies* then are detectable by an HIV test. Seroconversion may occur anywhere from days to weeks or months following HIV infection.
Septic shock: *Shock* resulting from infection and *sepsis*; can cause multiple organ failure and death; occurs most commonly in people with a compromised immune system.
Sepsis: A serious medical condition characterized by inflammatory reactions throughout the body resulting from the presence of an infection; can lead to *septic shock*, multiple organ failure, and death.
Shock: A serious, life-threatening medical emergency during which insufficient blood flow to the tissues results in lack of oxygen and nutrient supply; can be caused by various factors, such as trauma or an infection.
Splenium: The thickened *posterior* border of the *corpus callosum*.
Steatohepatitis: A type of liver disease characterized by inflammation of and fat accumulation in the liver.
Steatosis: The abnormal retention of lipids in the cells, indicating an impairment of normal *triglyceride* metabolism.
Striatum: A part of the brain that is involved in the planning of movement pathways as well as in other cognitive processes; in humans the striatum is activated by stimuli associated with reward, but also aversive, novel, unexpected, or particularly intense stimuli with high *salience;* includes several nuclei, including the *caudate nucleus*.
Subcortical: Located beneath the outer layer (i.e., *cortex*) of the largest portion of the brain (i.e., the cerebrum).
Synergistic: The effect when two or more agents act together to achieve a result that neither of the agents could have achieved by itself.
Tissue atrophy: Partial or complete wasting away of a tissue; can result from a variety of causes genetic events, poor nourishment, poor circulation, lack of use of the tissue).
Triglyceride: The chemical form in which most fat molecules exists in food as well as in the body; consist of glycerol and three fatty acids.
Tumor necrosis factor (TNF-α): A type of *cytokine* that promotes inflammatory responses, stimulates neutrophils and *macrophages,* induces fever, and induces *macrophages* to produce *cytokines*.
Viral load: Amount of virus in a given volume of fluid (e.g., blood); is a measure of the severity of infection.
Viremia: Presence of viruses in the blood.
Virion: A mature virus particle that exists freely outside a host cell.
Wasting: Unintentional substantial weight loss
White matter: A generic term for a collection of nerve cell fibers (i.e., *axons*) in the central nervous system.

